# Supplementary material for: AffyMAPSDetector: a software tool to characterize Affymetrix GeneChip™ expression arrays with respect to SNPs
Source: BMC Bioinformatics. 2007 Jul 30;8:276. doi: 10.1186/1471-2105-8-276 (PMC1959249; doi:10.1186/1471-2105-8-276)
Supplement: Additional file 13 — AffyMAPSDetector v1 source code. AffyMAPSDetector source code. [file 1471-2105-8-276-S13.zip › AffyMAPSDetector_v1_src/doc/index-files/index-4.html]

D-Index


|  |  |  |  |
| --- | --- | --- | --- |
| **Overview** \| Package \| Class \| Use \| **Tree** \| **Deprecated** \| **Index** \| **Help** | | |  |
| **PREV LETTER**   **NEXT LETTER** | **FRAMES**    **NO FRAMES**     **All Classes** |  |  |


A B C D E F G H I J L M N O P R S T V W X Y 

---


## **D**

**DataState** - class affychip.snpdetector.model.DataState.: Title: Probe SNP Detector **DuplicateRemover** - class affychip.snpdetector.util.DuplicateRemover.: Title: Probe SNP Detector **DuplicateRemover()** - Constructor for class affychip.snpdetector.util.DuplicateRemover: **dataModel** - Variable in class affychip.snpdetector.util.PmMmHeatmapViewerModel: **dectectSnpsFor(String, String)** - Method in class affychip.snpdetector.model.SnpDetector: THIS METHOD IS USED ONLY FOR EXPERIMENT AND TESTING. **delimiter** - Static variable in class affychip.snpdetector.model.GeneInfo: **delimiter** - Static variable in class affychip.snpdetector.model.SnpInfo: **detectSnpsFor(GeneInfo)** - Method in class affychip.snpdetector.model.OptimizedSnpDetector: This method accesses a gene with a given accession number (from GenBank) and locus-link-id online and searches NCBI snp database for existence of SNPs in this gene's nucleotide-sequence. **detectSnpsFor(GeneInfo)** - Method in class affychip.snpdetector.model.SnpDetector: This method accesses a gene with a given accession number (from GenBank) and locus-link-id online and searches NCBI snp database for existence of SNPs in this gene's nucleotide-sequence. **display()** - Method in class affychip.snpdetector.ui.StepOnePanel: Called when the panel is set. **display()** - Method in class affychip.snpdetector.ui.StepThreePanel: **display()** - Method in class affychip.snpdetector.ui.StepTwoPanel: **display()** - Method in class jwf.NullWizardPanel: Called when the panel is set. **display()** - Method in class jwf.WizardPanel: Called when the panel is set.

---


|  |  |  |  |
| --- | --- | --- | --- |
| **Overview** \| Package \| Class \| Use \| **Tree** \| **Deprecated** \| **Index** \| **Help** | | |  |
| **PREV LETTER**   **NEXT LETTER** | **FRAMES**    **NO FRAMES**     **All Classes** |  |  |


A B C D E F G H I J L M N O P R S T V W X Y 

---
